# Mental Health Disease or Preventable Problem? Australian Dog Trainers’ Opinions about Canine Separation Anxiety Differ with Training Style

**DOI:** 10.3390/ani10081393

**Published:** 2020-08-11

**Authors:** Trepheena Hunter, Diane van Rooy, Michelle McArthur, Sara Bennett, Jonathan Tuke, Susan Hazel

**Affiliations:** 1 Wild Things Veterinary Behaviour Services, Rosanna, VIC 3084, Australia; 2 Dog and Cat Behaviour Consultations, Sunbury, VIC 3429, Australia; drdianevanrooy@gmail.com; 3 School of Animal & Veterinary Sciences, The University of Adelaide, Roseworthy, SA 5371, Australia; michelle.mcarthur@adelaide.edu.au (M.M.); susan.hazel@adelaide.edu.au (S.H.); 4 Department of Clinical Sciences, College of Veterinary Medicine, North Carolina State University, Raleigh, NC 27607, USA; sara_bennett@ncsu.edu; 5 School of Mathematical Sciences, The University of Adelaide, Adelaide, SA 5005, Australia; simon.tuke@adelaide.edu.au

**Keywords:** dog, dog trainer, separation anxiety, trainer perceptions, separation related behaviour

## Abstract

**Simple Summary:**

Separation anxiety is common. Signs are seen when dogs are alone or separated from their owner, and include destructive behaviour, vocalising, restlessness, and house soiling. Many dog owners do not seek help from veterinarians but might see a trainer. The Australian dog training industry is not regulated. Trainers have a range of experience, education, and qualifications, and use a variety of techniques. We surveyed trainers’ opinions about separation anxiety and found significant differences between reward-based and balanced trainers. Reward-based trainers rated involvement of a veterinarian and use of medication as more important than balanced trainers. More balanced trainers reported that medication was rarely necessary in the cases that they saw. Half the reward-based trainers believed separation anxiety was preventable compared with 95% of balanced trainers. We conclude that opinions about separation anxiety vary between trainers using reward-based and balanced training. Trainers are not taught, expected, or legally allowed to diagnose anxiety disorders. This study found that balanced trainers were less likely to recommend involvement of veterinarians who can make a diagnosis and rule out other causes of observed behaviours. Understanding differences in trainer attitudes may help to improve communication between trainers and veterinarians to better support dogs with separation anxiety.

**Abstract:**

Separation anxiety is common. Many dog owners do not seek help from a veterinarian but might consult a trainer. The objective of this study was to investigate Australian trainers’ opinions about separation anxiety. An online survey was distributed via training organisations, resulting in 63 completed surveys. Descriptive statistics and Fisher’s exact tests were applied. Respondents were grouped into reward-based (*n* = 41) and balanced (*n* = 22) trainers. Most trainers (82.5%) used multiple methods to identify separation anxiety but only 7.9% referred to a veterinarian for diagnosis. Reward-based trainers ranked assistance from a veterinarian and owner’s willingness to try medication as more important than balanced trainers (*p* < 0.05). More balanced trainers reported that medication was rarely necessary in the cases they saw: 50% balanced compared with 4.9% reward-based trainers, with 95% CIs of [28.2, 71.8] and [0.6, 16.5], respectively. Almost all (95.5%) balanced trainers believed separation anxiety was preventable compared with 52.6% of reward-based trainers (*p* < 0.05). We conclude that opinions about separation anxiety varied between reward-based and balanced trainers. Trainers are not taught, expected, or legally allowed to diagnose anxiety disorders. This study showed that balanced trainers were less likely to recommend involvement of veterinarians who can make a diagnosis and rule out other causes of observed behaviours.

## 1. Introduction

Dogs are a social species who have undergone selection to live alongside humans as companions for many thousands of years. They are a popular pet and approximately 40% of Australian households own a dog [1]. Dogs generally enjoy a close social bond with their owners, but they are often expected to cope with long periods of time alone while their owners are away from home. However, not all dogs cope well when their owners are away and canine separation anxiety is common, with an estimated worldwide prevalence of 13–29% [2,3,4,5,6,7].

Separation anxiety is a collection of behaviours that occur only when dogs are left alone or separated from a significant person(s) and involves physiological and behavioural signs of distress [8]. Common signs include destructive behaviour, hypersalivation, house soiling (in a house-trained dog), vocalisation, and restlessness [9,10,11,12]. The terminology used in the scientific literature to describe this problem includes separation anxiety, separation related behaviour, separation anxiety syndrome, isolation anxiety, and attachment-related problems. No formal definitions currently exist for each term and the terms are often used interchangeably [4,9,13]. Trainers and pet owners commonly use diagnostic terms, such as separation anxiety, colloquially. Use of the term ‘separation anxiety’ by trainers can be problematic as it implies a diagnosed anxiety disorder and making a diagnosis is an “act of veterinary science”, which legally should be performed by a registered veterinarian [14]. However, the aim of this study was to understand the opinions of trainers and therefore followed their colloquial use of the term separation anxiety.

While there is a substantial body of literature about canine separation anxiety, there is currently no literature reporting dog trainers’ opinions. Dog training is not regulated in Australia; anyone can declare themselves a dog trainer and no formal qualification or experience is required. Dog trainers do not necessarily receive or seek training in differentiating between normal and abnormal behaviour; the focus may be on how to teach specific behaviours and cues. Some certification courses for dog trainers teach the differences between normal and abnormal behaviour while others do not; some trainers seek out further education while others do not. As such, there is variation in the depth of knowledge within the dog training industry. Although trainers should not make diagnoses and should not be expected to distinguish between normal and abnormal behaviours, they are often the first to be consulted when pet owners have a problem. Many owners either do not seek help or delay seeking help from a veterinarian [7,15]. Owners may have an existing relationship with a dog trainer, through puppy school classes or adult dog obedience classes, or they may seek a trainer, believing that the problem is best addressed by a trainer rather than a veterinarian. As such, trainers may be the first to provide management advice to owners. It is therefore important to understand dog trainers’ understanding and management of separation anxiety.

Dog training usually involves learning via both classical and operant conditioning; these processes are explained in detail in both academic and professional texts [16,17,18]. There are no formal definitions of dog training styles and there are many terms used. Trainers who identify as “reward based”, “positive reinforcement”, “force-free” or “least invasive, minimally aversive” use predominantly positive reinforcement to teach new behaviours. They may use some negative reinforcement and negative punishment but avoid use of positive punishment. Some trainers use positive and negative reinforcement as well as positive and negative punishment. In Australia, trainers who take this approach generally refer to themselves as “balanced”; this paper therefore also uses the term “balanced” for trainers who use positive punishment as well as positive and negative reinforcement techniques. Trainers identifying themselves as “traditional trainers” tend to use predominantly positive punishment techniques.

There is no official registry for dog trainers. It is not possible to accurately determine the number of dog trainers practicing in Australia, nor is it possible to determine the breadth of training styles used. The number of trainers can be estimated, as some dog training organisations list their members online. This will not be accurate as dog trainers may belong to more than one organisation, they may not belong to any organisation, or they may be a member but not be listed online. The main dog training organisations in Australia are Association of Pet Dog Trainers Australia (426 members listed), Pet Professional Guild Australia (187 members listed), Delta Society (78 members listed), National Dog Trainers Federation (25 members listed) and the Australian Association of Professional Dog Trainers (9 dog training businesses listed) [19,20,21,22,23].

General practice veterinarians have a range of knowledge and experience in veterinary behavioural medicine that depends on the education they received during veterinary school and their choice of continuing education. Some veterinarians have extensive knowledge of behavioural medicine while others have more limited knowledge. In Australia, the term “veterinary behaviourist” refers specifically to a registered specialist in veterinary behavioural medicine. However, veterinarians who work in the field of behavioural medicine are often called “veterinary behaviourists” by trainers and the general public regardless of their qualifications. This study was aimed at trainers and therefore the term “veterinary behaviourist” was used broadly to refer to any veterinarian working in the field of veterinary behavioural medicine.

The present study aimed to investigate Australian dog trainers’ opinions about the management of canine separation anxiety. We hypothesised that opinions would differ between trainers using different training methods.

## 2. Materials and Methods

### 2.1. Human Ethics

The protocols and questionnaire used in this study were approved by the University of Adelaide Human Research Ethics Committee (H-2016-012).

### 2.2. Questionnaire

Since there were no existing validated survey instruments to use, the questionnaire was developed through existing literature and discussions with dog trainers who worked with dogs with separation anxiety, and then tested by piloting the questions with the same trainers. The online questionnaire contained 31 questions over 4 sections, detailed in Appendix A. Only questions that contributed meaningfully to the aim of this study are included, and open-ended questions have also been excluded from the paper. Questions included are: Section A included 8 questions concerning demographic information; Section B contained 6 questions concerning prevalence and diagnosis; Section C included 2 questions on the perceptions of contributing factors; and Section D contained 3 questions on the management of separation anxiety. The questionnaire was available online from June to November 2016.

Section B asked trainers about the extent to which they encountered specific behaviours that may be associated with separation anxiety (see Appendix A). The behaviours included in the list were selected based on the commonly reported signs of separation anxiety [10,11,12,24,25]. The question did not ask respondents to explain how these behaviours were assessed, for example obtaining video footage or interviewing owners.

### 2.3. Participants

Participants were recruited initially via known dog training organisations. These organisations were sent an information sheet, a link to the online questionnaire, and a request to invite trainers to participate. Snowballing recruitment was used, with people passing on the survey using social media, to reach as many and as wide a population of dog trainers as possible. Inclusion criteria were that participants were “dog trainers”, defined as adults who provide any service to dog owners or handlers in Australia that claims to modify a dog’s behaviour. Veterinarians who did part-time dog training or provided behaviour modification exercises as part of their treatment protocol were excluded.

Respondents were classified into two groups—reward-based and balanced trainers. This grouping was based on the respondents’ description of their training style. The survey included the options force-free dog training, alpha dog/dominance obedience training, balanced (both reward based/dominance obedience), or other. Those who selected “other” wrote a free-text description of their training style. The reward-based group included respondents who selected the force-free dog training option and those who wrote “positive reinforcement”, “least intrusive, minimally aversive”, or described their style as using positive reinforcement techniques in the free-text response. The balanced group included those who selected the balanced option and those who explained that they used positive and negative reinforcement and positive and negative punishment in their training approach. The responses of all respondents fell into these two groups.

### 2.4. Statistical Analysis

After the survey was closed, the dataset was downloaded into an Excel spreadsheet for data management. For the question about the methods used to confirm separation anxiety, an additional category “referral to a veterinarian” was created to capture a common response written in “other”. The data was read into R version 4.0.2 [26] and cleaned. All Likert-type responses were treated as categorial variables. The mode was calculated for the Likert-type responses, identifying the most frequently recorded response. There were insufficient observations to perform a logistic regression with multiple predictors. Fisher’s exact tests were run for each predictor. Given the number of tests performed, the *p*-values were adjusted with a false-discovery-rate (FDR) adjustment. An FDR-adjusted *p*-value of <0.05 was taken to indicate statistical significance. The 95% confidence interval was calculated for questions relating to the proportion of respondents in each group.

## 3. Results

### 3.1. Response Rate

One hundred and forty one surveys were submitted. Seven surveys were completed by veterinarians rather than trainers and were excluded. Seventy-one surveys were excluded due to incomplete data: 30 contained no data, 39 contained demographic answers only, and 2 included less than 75% response. The results of 63 surveys were included in the data analysis for Sections A to C. Results of 60 surveys were included in the analysis of Section D, with 3 surveys removed due to incomplete responses to Section D questions.

### 3.2. Reward-Based or Balanced Trainers

There were 22 balanced and 41 reward-based respondents. For Section D, there were 22 balanced and 38 reward-based respondents. No respondents selected alpha dog/dominance or wrote a free-text response indicating that punishment was their primary training method.

### 3.3. Demographics

Respondents were mainly female (61 of 63 respondents) and ranged from 18 to 24 to over 65 years of age. Table 1 shows respondent demographics.

In the reward-based group, nearly all (95.1%) respondents had completed vocational or tertiary education programs compared to 63.6% in the balanced group. More respondents in the balanced group listed their education level as Year 12 or less than those in the reward-based group (36.4% balanced; 4.9% reward based). More than half the respondents listed a Certificate III or IV in Companion Animal Services/Dog Behaviour or equivalent at their highest certification in dog training. There were no statistically significant differences in age, education, or certification between the two groups, with FDR-adjusted *p*-values of 0.363, 0.057, and 0.783, respectively.

Years worked as a trainer ranged from 0.3 to 35 years. The median number of years worked was higher for the reward-based group (9 years) than the balanced group (6 years); the years worked did not differ significantly between the groups (FDR-adjusted *p*-value = 1). The two groups spent similar proportions of work time in puppy school classes, dog training classes, and one on one consultations (FDR-adjusted *p*-values of 1, 0.951, 0.882 respectively). Grouped together, respondents spent the highest percentage of work time doing one on one consultations (54.8%) with 23.8% of time in dog training classes and 16.2% in puppy school classes.

### 3.4. Prevalence and Diagnosis of Separation Anxiety

Respondents reported seeing between 0 and 5+ cases of separation anxiety in the past month with 1–2 cases most frequently reported (53.6% reward based, 40.9% balanced). The number of cases seen per month did not differ significantly between the two groups (FDR-adjusted *p*-value = 0.462).

The frequency of separation anxiety signs seen in various situations is shown in Table 2. Both groups of respondents reported that they most commonly saw dogs with separation anxiety show signs of stress or anxiety when left alone with no human or dog company. There were no significant differences between the two groups.

The signs of separation anxiety reported by trainers are shown in Table 3. There were no statistically significant differences in the reported frequency of signs of separation anxiety between the two groups.

One respondent used only a single method to confirm suspected signs of separation anxiety while 82.5% of respondents used three or more methods. Table 4 shows the different methods used by trainers to confirm signs of separation anxiety. Only five respondents, all from the reward-based group, referred to a veterinarian for diagnosis of separation anxiety.

Reasons cited for not referring a client to a veterinarian or veterinary behaviourist for treatment are shown in Table 5. The balanced group were more likely to select “rarely necessary for medication in the cases I see”: 50.0% balanced compared with 4.9% reward-based respondents, with 95% CI of [28.2, 71.8] for balanced and [0.6, 16.5] reward based indicative of a significant difference.

Both groups ranked “owner compliance” as important or extremely important to the success of managing separation anxiety (Table 6). The reward-based trainer respondents ranked the following higher than the balanced trainer respondents with FDR-adjusted *p*-value < 0.05: assistance from a veterinary behaviourist, assistance from a veterinarian, and owner’s willingness to try medication.

### 3.5. Factors Contributing to Development of Separation Anxiety

Situations that trainers associate with the onset of separation anxiety are shown in Table 7, with the highest frequency reported for “after rehoming” and “following a household change” by both groups of respondents. There were no significant differences between the two groups.

Ranking of factors that respondents reported would contribute to the development of separation anxiety are shown in Table 8. There were no significant differences between the two groups.

### 3.6. Management of Separation Anxiety

The importance of specific factors in the management of separation anxiety is shown in Table 9. The reward-based group ranked three factors significantly higher than the balanced group (FDR-adjusted *p*-value < 0.05): referral for medication, not leaving the dog alone, and dog’s access inside when alone. The reward-based group ranked owner–dog training interactions significantly lower than the balanced group (FDR-adjusted *p*-value = 0.024).

There was a significant difference in opinion about whether separation anxiety can be prevented: 52.6% reward-based trainers replied “yes” compared with 95.5% of balanced trainers (FDR-adjusted *p*-value = 0.032). Most respondents (84.2% reward based, 90.5% balanced) reported that they could sometimes identify a puppy (less than 6 months) who may develop separation anxiety.

## 4. Discussion

To our knowledge, this is the first survey of dog trainers’ perceptions and management of canine separation anxiety. We found that trainers’ views differed significantly with their style of training. Most striking was the difference in opinions about medication and involvement of veterinarians. Reward-based trainers consistently ranked use of medication and veterinarian involvement significantly higher than balanced trainer respondents. When asked how important assistance from a veterinary behaviourist was in the success of management of separation anxiety, the most frequently reported response was “extremely important” from reward-based trainers compared with “not important” from balanced trainers. Similarly, reward-based trainers rated referral for medication significantly higher than balanced trainers. This was not explained by differences in demographics, experience, or case identification, as there were no significant differences between the groups in demographic variables, years of experience as a trainer, number of cases of separation anxiety seen per month, or frequencies of signs of separation anxiety reported. Rather, there may be a different understanding of separation anxiety; reward-based trainers may be more likely to view separation anxiety as an affective disorder, or mental health problem, for the dog rather than a training issue.

The notable difference between the groups was further demonstrated by the response seen when trainers were asked why they would choose not to refer to a veterinarian or veterinary behaviourist. Half of the balanced trainers felt medication was “rarely needed in the cases they saw” compared to a small proportion (4.9%) of reward-based trainers. Current scientific literature shows that psychotropic medications are an effective component of separation anxiety treatment, together with management strategies and behaviour modification [10,27,28,29,30,31,32,33,34]. If trainers are concerned about use of medication, it is likely that owners will be reluctant to seek veterinary treatment: pet owners are likely to be guided by their trainer’s beliefs. Trainers do not necessarily have any formal education in recognising affective disorders, and it would not be appropriate to expect trainers to determine when medication may be indicated. It is therefore concerning that a number of trainers in this study felt confident to decide that medication was not indicated. This highlights two points: firstly, the importance of ongoing education for trainers on the potential for an affective basis for behaviour problems; and secondly, the need to encourage collaborative care between trainers, veterinarians, and other qualified animal behaviour professionals to most effectively support the dog and owner.

The groups differed significantly when asked whether separation anxiety was preventable: almost all balanced trainers believed it was preventable compared with half of the reward-based trainers. If separation anxiety were to be preventable, the aetiology should involve only factors that relate to management and training and not those with an affective basis. The aetiology of separation anxiety is not yet clearly defined and appears to be multifactorial, including factors beyond those related to management and training. Hyperattachment has been proposed as a contributing factor [35] but some authors argue that it is neither definitive nor necessary for diagnosing separation anxiety [8,13]. A recent study by de Assis et al. [9] assigned dogs with separation related problems to four clinically distinct clusters, suggesting that there may be several aetiologies for separation anxiety. Genetic links have been shown in many human psychiatric conditions [36,37,38] and the potential for a genetic association in canine separation anxiety has been proposed. Several candidate genes for separation anxiety have been identified and an association with separation anxiety has been demonstrated for one gene in a cohort of Golden Retrievers [39]. Several studies have examined risk factors associated with separation anxiety and the data is equivocal. Source of acquisition is associated with the prevalence of separation anxiety, with dogs acquired from shelters and pet shops showing a higher prevalence than dogs from breeders or friends [40,41]. The findings on sex are inconsistent, with some studies showing an increased prevalence in males [7,24], while others found no association with sex [41]. There is no consensus on predisposed breeds: some authors have reported predisposition in particular breeds [24,40,41], while others report no breed predisposition [42]. Given that the aetiology is complex, multifactorial, and incompletely understood, it is difficult to categorically conclude that separation anxiety is preventable. The striking difference between reward-based and balanced trainers in this study may reflect trainers’ understanding of behavioural development, including the role of genetics, environment, social relationship development, external stressors, and learning [43]. The balanced trainer respondents ranked training as more important for separation anxiety management than the reward-based respondents. It is possible that the balanced trainer respondents in this study felt that the owner’s behaviour and management caused separation anxiety and therefore that separation anxiety could be prevented by altering human behaviour. Further work is required to understand why trainers who use particular training techniques have a different understanding of separation anxiety. This could inform development of continuing education resources for trainers and help trainers, veterinarians, and other animal behaviour professionals to work collaboratively.

The signs of separation anxiety reported in this survey are in agreement with previous studies [10,11,12,24,25,35]. This suggests that trainers were observing the behavioural and physiological signs commonly seen with separation anxiety [44,45], although respondents were not asked to describe how these behaviours and physiological signs were observed. While these signs occur in dogs with separation anxiety, they are non-specific and diagnosis involves excluding differential diagnoses [44,45,46]. There are many differential diagnoses including behavioural and medical conditions which may not be immediately apparent. For example, there is report of comorbidity between separation anxiety and noise phobia [47] and noise phobia may be comorbid with musculoskeletal pain [48]. A recent study also reported a high prevalence of pain in dogs seen in specialist behaviour practices [49]. Trainers reported seeing signs of distress when dogs were able to see their owners but unable to access them. While this is often observed in dogs with separation anxiety, it may also indicate other problems such as confinement distress [46]. Few respondents considered referral to a veterinarian or veterinary behaviourist for diagnosis. There may be both a lack of understanding of potential medical causes for behaviour problems and a misunderstanding of the role of a veterinarian in animal care, particularly in the area of behaviour. The Australian Veterinary Association defines “acts of veterinary science” as services which form part of the practice of veterinary surgery and medicine, which includes the diagnostic confirmation of, treatment of, and provision of management advice for infectious disease, physiological dysfunction, psychological dysfunction, and injury in animals [50]. Making a diagnosis of separation anxiety is an “act of veterinary science”, which legally should be performed by a veterinarian [14]. Veterinarians have an important role, ensuring that concurrent medical conditions are either ruled out or identified and treated in order to give the best prognosis for successful treatment.

This study had several limitations including self-selected respondents and low number of male respondents. The respondents all identified as either reward-based or balanced trainers; none of the respondents identified as dominance-based, traditional, or aversive trainers. This may not accurately reflect the training community. There is no information available on the techniques used by Australian dog trainers, but it would be naïve to assume that there are no trainers using dominance-based or aversive training techniques. Trainers answered the survey questions based on their previous experience and opinions about dogs showing separation anxiety. As discussed earlier, there is a range of terminology used for the behaviours seen when dogs are alone and there are no accepted definitions for these terms. It is possible that respondents in this study understood “separation anxiety” differently. Given that the dogs that they worked with may not have received a diagnosis, it is possible that respondents were referring to dogs experiencing a range of behavioural motivations, including anxiety, hyperattachment, frustration, and boredom. Section B of the questionnaire used the term diagnosis (“Prevalence and Diagnosis”). This may have implied that it was appropriate for trainers to make a diagnosis, however the term was not used in any of the questions.

This study had a low number of respondents and high variance in the data, which prevented use of a logistic regression with multiple predictors (due to low statistical power) and some correlations may have been missed (type II error). Future studies with a larger number of respondents, including trainers who used predominantly positive punishment techniques, are recommended. The data was collected over a six-month period so there is a potential for missing seasonal trends in case load or presentation.

Detailed analysis of training style and trainers’ knowledge of learning theory was beyond the scope of this study. There is likely to be variation in the techniques applied and the proportions of positive reinforcement and positive punishment used by the balanced trainers and, perhaps to a lesser degree, the reward-based trainers. Further investigation may offer insight into the differences observed in the present study. Future studies determining where and what trainers learn about behaviour problems, such as separation anxiety, would also inform the development and provision of evidence-based continuing education resources for dog trainers.

## 5. Conclusions

This study investigated the opinions of reward-based and balanced dog trainers about canine separation anxiety. Dog trainers from both groups reported the same signs of separation anxiety that are reported in the literature. There were several notable differences between the groups. Reward-based trainers ranked involvement of veterinarians and owners’ willingness to try medication as significantly more important than the balanced group. Balanced trainers were more likely to report that medication was rarely necessary for the cases of separation anxiety that they saw and to believe that separation anxiety can be prevented. While trainers are not taught, expected, or legally allowed to make a diagnosis of separation anxiety, this study indicates that balanced trainers are less likely to recommend the involvement of a veterinarian when managing dogs with suspected separation anxiety. This has the potential to delay diagnosis and impact treatment outcome if any concurrent diagnoses are not identified. Appreciation of the differences reported in this study between reward-based and balanced trainers may be used to improve communication and teamwork between all groups working to support the mental health of dogs.

## Figures and Tables

**Table 1:** Demographics (absolute number and percentage) for the reward-based trainer (*n* = 41) and balanced trainer (*n* = 22) respondents.

Demographic	Variable	Reward Based (%)	Balanced (%)
Age (years)	18–24	0 (0.0)	2 (9.1)
	25–34	10 (24.4)	4 (18.2)
	35–44	9 (21.9)	11 (50.0)
	45–54	11 (26.8)	3 (13.6)
	55–64	7 (17.1)	2 (9.1)
	Over 65	4 (9.8)	0 (0)
Education	Year 12 or less	2 (4.9)	8 (36.4)
	Vocational or diploma	21 (51.2)	8 (36.4)
	Bachelor or post-graduate	18 (43.9)	6 (27.3)
Certification	Workshops/informal courses	2 (4.9)	1 (4.6)
	Certificate III or IV ^1^	28 (68.3)	11 (50.0)
	Certified professional dog trainer ^2^	8 (19.5)	5 (22.7)
	Diploma or Bachelor in Dog Behaviour ^2^	1 (2.4)	1 (4.6)
	Other	2 (4.9)	4 (18.2)

^1^ Certificate III or IV in Companion Animal Services, Dog Behaviour, or equivalent; ^2^ or equivalent.

**Table 2:** Frequency of separation anxiety signs (mode and range of rank) in different situations as reported by reward-based trainer (*n* = 41) and balanced trainer (*n* = 22) respondents.

Situation	Reward Based	Balanced	FDR-Adjusted *p*-Value
Left alone (no human/dog company)	5 (2–5)	4 (2–5)	0.840
Owner prepares to leave	4 (1–5)	3 (2–5)	0.366
Prevented access but can see owner	3 (1–5)	4 (2–5)	0.277
In presence of owner, always trying to be in proximity for physical contact	3 (1–5)	3 (1–5)	1
Away from home	2 (1–5)	3 (1–4)	0.913
Away from owner but people/dog present	2 (2–5)	3 (2–4)	0.394

Ranking 1–5 based on Likert-type scales (1 = never; 5 = always).

**Table 3:** Signs of separation anxiety (mode and range of rank) reported by reward-based trainer (*n* = 41) and balanced trainer (*n* = 22) respondents.

Situation	Reward Based	Balanced	FDR-Adjusted *p*-Value
Excessive vocalisation	4 (2–5)	4 (3–5)	0.825
Excessive greeting behaviours	4 (2–5)	4 (3–5)	0.372
Attempts to remain close to owner at all times	4 (2–5)	4 (3–5)	0.882
Escaping or escape attempts	4 (1–5)	4 (2–5)	0.951
Excessive departure behaviours	4 (1–5)	4 (1–5)	0.882
Excessive panting/other respiratory signs	4 (1–5)	4 (2–5)	0.840
Ritualised/repetitive behaviours (pacing, circling)	4 (1–5)	4 (2–5)	0.882
Changes in eating/drinking habits	4 (1–5)	3 (1–4)	0.692
Destructive behaviours	3 (2–5)	4 (2–5)	0.312
Hypervigilance or hyperalertness	3 (2–5)	4 (1–5)	0.062
Trembling/shaking	3 (1–5)	4 (1–5)	0.825
Increased reactivity in other situations (exposure to new situations, social encounters)	3 (2–5)	4 (1–4)	0.783
Excessive salivation	3 (1–5)	3 (2–4)	0.913
House-soiling (urination/defecation)	3 (1–5)	2 (1–4)	0.956
Increased aggression behaviours (toward other dogs, humans)	3 (1–5)	2 (1–4)	0.783
Increased grooming, possible self-mutilation	3 (1–5)	2 (1–4)	0.851
Vomiting/diarrhoea	2 (1–5)	2 (1–4)	1
Immobility/freezing or decreased activity	2 (1–5)	2 (1–3)	1

Ranking 1–5 based on Likert-type scales (1 = never; 5 = always).

**Table 4:** Methods used to confirm suspected separation anxiety by reward-based trainer (*n* = 41) and balanced trainer (*n* = 22) respondents.

Method Used	Reward Based		Balanced	
	%	95% CI	%	95% CI
Owner reports	85.4	[70.8, 94.4]	90.9	[70.8, 98.9]
Observation of dogs in class	36.6	[22.1, 53.1]	45.5	[24.4, 67.8]
Observation of dogs at home	82.9	[67.9, 92.8]	90.9	[70.8, 98.9]
Functional assessment	43.9	[28.5, 60.3]	50.0	[28.2, 71.8]
Video footage	68.3	[51.9, 81.9]	54.5	[32.2, 75.6]
Activity monitor	9.8	[2.7, 23.1]	4.5	[0.1, 22.8]
Refer to veterinarian	14.6	[5.6, 29.2]	0.0	[0.0, 15.4]

CI: confidence interval.

**Table 5:** Reasons for choosing not to refer to a veterinarian or veterinary behaviourist for treatment given by reward-based trainer (*n* = 41) and balanced trainer (*n* = 22) respondents.

Reason	Reward Based		Balanced	
	%	95% CI	%	95% CI
Medication rarely necessary in the cases I see	4.9	[0.6, 16.5]	50.0	[28.2, 71.8]
Always refer when required	73.2	[57.1, 85.8]	36.4	[17.2, 59.3]
Owner finances	58.5	[42.1, 73.7]	40.9	[20.7, 63.6]
Owner reluctant to medicate	51.2	[35.1, 67.1]	40.9	[20.7, 63.6]
Limited access	29.3	[16.1, 45.5]	9.1	[1.1, 29.2]
Veterinarian uncooperative	14.6	[5.6, 29.2]	13.6	[2.9, 34.9]
Veterinary behaviourist uncooperative	4.9	[0.6, 16.5]	13.6	[2.9, 34.9]

CI: confidence interval.

**Table 6:** Factors (mode and range of rank) that affect the success of management of separation anxiety cases according to reward-based trainer (*n* = 41) and balanced trainer (*n* = 22) respondents.

Factor	Reward Based	Balanced	FDR-Adjusted *p*-Value
*Factors ranked higher by reward-based trainers*			
Assistance from veterinarian behaviourist	5 (1–5)	2 (1–5)	0.011
Assistance from veterinarian	4 (3–5)	4 (1–4)	0.011
Owner’s willingness to try medication	4 (2–5)	2 (1–5)	0.011
*No significant difference in ranking between groups*			
Owner’s compliance	5 (4–5)	5 (4–5)	0.913
Owner’s availability for owner–dog interactions	5 (1–5)	5 (3–5)	0.882
Owner’s attitude to discipline	5 (1–5)	4 (2–5)	0.825
Owner’s level of stress	4 (2–5)	5 (3–5)	0.882
Dog’s stimulation when left	4 (1–5)	5 (3–5)	0.882
Owner’s social support (friends/family willing to help)	4 (3–5)	4 (2–5)	0.103
Owners view of mental health	4 (3–5)	4 (2–5)	0.261
Dog’s access to inside house when left	4 (2–5)	4 (1–5)	0.079
Dog’s temperament	4 (1–5)	4 (4–5)	0.462
Owner’s financial situation	4 (3–5)	3 (1–5)	0.051
Crate training	4 (1–5)	4 (1–5)	0.390
Dog’s age	4 (1–5)	4 (1–5)	0.920
Dog’s breed	3 (1–5)	3 (1–5)	0.951

Ranking 1–5 based on Likert-type scales (1 = extremely not important; 5 = extremely important).

**Table 7:** Factors (mode and range of rank) associated with the onset of separation anxiety as reported by reward-based trainer (*n* = 41) and balanced trainer (*n* = 22) respondents.

Factor	Reward Based	Balanced	FDR-Adjusted *p*-Value
After rehoming (includes shelter, adopted dogs)	4 (1–5)	4 (3–5)	0.312
Following a household change (house move, new baby)	4 (2–4)	4 (2–4)	1
Following a negative event whilst left alone	3 (1–4)	4 (2–5)	0.783
Old age (aging elderly dogs)	3 (1–5)	3 (1–4)	0.851
Puppyhood	2 (1–4)	3 (1–4)	0.882

Ranking 1–5 based on Likert-type scales (1 = never; 5 = always).

**Table 8:** Factors (mode and range of rank) that reward-based trainer (*n* = 41) and balanced trainer (*n* = 22) respondents report to contribute to the development of separation anxiety.

Factor	Reward Based	Balanced	FDR-Adjusted *p*-Value
Owner’s response to attention-seeking dog behaviour	5 (3–5)	5 (4–5)	0.290
Owner’s approach to departure/greeting rituals	5 (2–5)	5 (4–5)	0.783
Owner’s attitude to training/discipline	5 (1–5)	5 (4–5)	0.882
Gradual exposure to separation	5 (2–5)	5 (2–5)	1
Level of exercise (not enough or too much)	4 (1–5)	5 (3–5)	0.312
Dog’s environment when left alone	4 (3–5)	5 (3–5)	0.905
Type of owner–dog interactions	4 (2–5)	4 (4–5)	1
Owner’s time spent with dog (always/never with dog)	4 (3–5)	4 (3–5)	0.882
Dog’s temperament	4 (2–5)	4 (2–5)	1
Dog’s genetics	4 (3–5)	4 (2–5)	0.783
Number of homes	4 (2–5)	4 (2–5)	0.676
Age of acquisition	4 (2–5)	4 (2–5)	0.951
Source of acquisition (e.g., shelter)	4 (1–5)	3 (2–5)	0.585

Ranking 1–5 based on Likert-type scales (1 = extremely not important; 5 = extremely important).

**Table 9:** The importance (mode and range of rank) of specific factors when managing separation anxiety according to reward-based trainer (*n* = 38) and balanced trainer (*n* = 22) respondents.

Factor	Reward Based	Balanced	FDR-Adjusted *p*-Value
*Factors ranked higher by reward-based trainers*			
Referral for medication	4 (3–5)	2,4 (1–5)	0.011
To not leave dog alone (e.g., access dog sitters)	4 (1–5)	3 (1–4)	0.011
Dog’s access to inside house when left alone	4 (3–5)	3 (1–5)	0.028
*Factors ranked higher by balanced trainers*			
Owner–dog structured interactions (e.g., training)	4 (3–5)	5 (3–5)	0.024
*No significant difference in ranking between groups*			
Behaviour modification—graded exposure to separation	5 (3–5)	5 (2–5)	0.882
Owner’s ability to tune into dog’s emotions (stressed/happy)	5 (4–5)	5 (4–5)	1
Encouraging dog’s confidence/independence	5 (4–5)	5 (4–5)	0.783
Desensitisation of dog to owner’s departure cues	5 (3–5)	5 (4–5)	0.882
Owner’s attitude to discipline/training	4 (1–5)	5 (4–5)	0.783
Owner behaviour toward attention-seeking behaviours	4 (3–5)	5 (4–5)	0.168
Dog’s stimulation when left	4 (3–5)	4 (2–5)	0.372
Relaxation exercises (e.g., massage)	4 (2–5)	4 (2–5)	0.693
Use of therapeutic agents (e.g., pheromone collar)	4 (2–5)	3 (2–5)	0.394
Dog’s level of exercise	4 (1–5)	5 (2–5)	0.363
Owner–dog play (e.g., unstructured interactions)	3 (3–5)	4 (3–5)	0.676
Increase predictability of routines	3 (1–5)	3 (1–5)	1
Decrease predictability of routines	3 (1–5)	4 (2–5)	0.882

Ranking 1–5 based on Likert-type scales (1 = extremely not important; 5 = extremely important).

## Appendix A. Australian Dog Trainers’ Perspectives and Management of Separation Anxiety in Pet Dogs Questionnaire

Questions that were excluded from the final analysis are given in italics.

**Section A: Demographic information**
Gender: male/female/other Age: 18–24; 25–34; 35–44; 45–54; 55–64; 65+ Education level: Less than Year 12; Year 12 or equivalent; vocational or diploma (Associate/Undergraduate) qualification; Bachelor’s degree (including Honours); Postgraduate/Masters/Doctorate diploma What is your highest certification level in dog training: no formal qualifications; attendance to workshops and informal courses; Certificate III or IV Companion Animal Services/Dog Behaviour or equivalent; Certified professional dog trainer or equivalent; Other (please specify) How many years have you worked as a dog trainer Which of the following categories best describes your dog training work: full time, part time, other (please specify) What percentage (0–100%) of your work time consists of: puppy preschool classes; dog training classes; one-on-one consultations What style of training do you believe to be most effective: force-free dog training; alpha dog/dominance obedience training; balanced; other (please specify) Are you affiliated with a dog training club and/or professional body? If so please specify below (optional) If you belong to a dog training club/organization how many other trainers do you work with? (optional)

**Section B: Prevalence and diagnosis**
What are the ages of dogs you regularly work with? (you may choose more than one): <6 months, 6 months to <1 year, 1 year to <3 years, 3+ years How old are the dogs you see with separation anxiety? (you may choose more than one): <6 months, 6 months to <1 year, 1 year to <3 years, 3+ years In the last month how many cases of separation anxiety have you worked with: 1–2, 3–4, 5+ What percentage of the dogs you see with separation anxiety struggle to cope when: left alone (no human/dog company); away from owner but people/dog present; prevented access but can see owner; owner prepares to leave; away from home or a place; in the presence of owner but always tries to be in touching distance of owner (never, occasionally, sometimes, frequently, always) Rate the extent to which you encounter the following symptoms in dogs who show signs of separation anxiety (never, occasionally, sometimes, frequently, always): destructive behaviour; inappropriate urination/defecation; excessive vocalisation; excessive salivation; excessive panting/other respiratory signs; trembling/shaking; immobility/freezing or decreased activity; escaping or escape attempts; hypervigilance or hyper-alertness; evidence of gastrointestinal disorders (vomiting/diarrhoea); changes in eating/drinking habits; increased grooming, possible self-mutilation; ritualised/repetitive behaviours (pacing, circling); excessive departure behaviours; excessive greeting behaviours; attempts to maintain close proximity to owner at all times; increased reactivity in other situations (exposure to new situations, social encounters); increased aggression behaviours (toward other dogs, humans) What methods do you use to confirm suspected signs of separation anxiety: owner self-reports; behavioural observations of dogs and owners at class; behavioural observations of dogs and owners on home visits; functional assessment (leading to a contingency statement and behavioural modification program); video footage of dog’s behaviour during owner absence; activity monitor sensors; other (please specify) What may prevent you from referring to a veterinarian or veterinary behaviourist for treatment: limited access (not one available in your area); owner reluctance to involve medications; limited finances of owner; rarely necessary for medication in the cases I see; lack of cooperation from veterinarian; lack of cooperation from veterinary behaviourist; always refer when required; other (please specify) From your experience how important are the following factors in influencing the success of managing separation anxiety cases (extremely not important, not important, neutral, important, extremely important): breed; age; temperament; access to inside house when left; crate training; stimulation when left; owner’s time availability for owner–dog interactions; owner’s willingness to try medication; owner’s attitude to discipline; owner’s compliance; owner’s level of stress; owner’s level of social support (friends/family willing to help with intervention); owner’s financial situation; owner’s view of mental health; assistance from veterinarian; assistance from veterinary behaviourist.

**Section C: Perceptions of contributing factors and coinciding conditions**
In your experience rate the extent (never, occasionally, sometimes, frequently, always) to which you see the following conditions in the dogs you work with showing signs of separation anxiety: anxiety in other situations (e.g., being outside or away from home, interactions with strangers, other dogs); fear of thunderstorms; fear of loud noises (excluding thunder storms) e.g., fireworks; medical condition (skin condition, allergies, gastrointestinal disorders); aggression (related to food/handling/strangers/other dogs); other (please specify) Separation anxiety symptoms can emerge at different times in a dog’s life. Rate the extent (never, occasionally, sometimes, frequently, always) of cases you see with the onset of separation anxiety symptoms from: puppyhood; following a negative event whilst left alone; following a household change (house move, new baby); after rehoming (includes shelter, adopted dogs); old age (aging elderly dogs) From your experience, rate the importance of the following as potential contributing factors in the development of separation anxiety (extremely not important, not important, neutral, important, extremely important): source of acquisition (e.g., shelter); genetics; number of homes; age of acquisition; gradual exposure to separation; owner’s time spent with dog (always or never with the dog); type of owner–dog interactions; owner’s behaviour/responses to attention-seeking behaviour; owner’s approach to departure/greeting rituals; owner’s attitude to training/discipline; dog’s temperament; dog’s environment when left alone; level of exercise (not enough or too much); other (please specify) In your experience from the above options provide the top three factors you believe contribute to the development of separation anxiety symptoms in pet dogs

**Section D: Management of separation anxiety of pet dogs**
In your experience when managing cases of separation anxiety what is the degree of importance you attribute to the following factors (extremely not important, not important, neutral, important, extremely important): dog’s stimulation when left; dog’s access to inside house when left; dog’s behaviour modification (graded exposure to separation); desensitisation of dog to owner’s departure cues; encouraging dog’s confidence/independence; owner’s attitude to discipline/training; owner’s ability to tune into dog’s emotions (identify stressed vs. happy dog); use of therapeutic agents (e.g., Adaptil collar); referral for medication; dog’s level of exercise; increase predictability of routines; decrease predictability of routines; owner’s behaviour toward dog’s attention-seeking behaviours; owner–dog structured interactions (e.g., training); relaxation exercises (e.g., massage); to not leave dog alone (e.g., access dog sitters); owner–dog play (e.g., unstructured interactions); other (please specify) In the last month how many cases of separation anxiety did you refer to a veterinarian? In the last month how many cases of separation anxiety did you refer to a veterinary behaviourist? From your experience how to you rate the importance of the following areas to attempt to change when treating separation anxiety in pet dogs (extremely not important, not important, neutral, important, extremely important): environmental enrichment; improve dog’s ability to cope with being left (e.g., medication); owner’s attitude and behaviour; owner–dog interactions Do you believe separation anxiety in pet dogs can be prevented? yes, no, unsure If you answered yes or unsure to the previous question, select any of the following you believe to assist in the prevention of separation anxiety in pet dogs: gradual exposure to separation from owner; providing environmental enrichment when left; socialisation – exposure to wide range of events during early development; encouraging the puppy to engage in independent behaviours; reduce owner fuss during departure/greeting; providing comfortable/safe place whilst left; other (please specify) How often do you use the following to monitor the progress of treatment interventions (never, occasionally, sometimes, usually, always): verbal self-reports of owners; direct behavioural observations; neighbour reports; ongoing functional assessment; video footage of dog’s behaviour during owner absence; activity monitor sensors; owner diaries of dog activities; not applicable (do not monitor progress after initial consultation); other (please specify) In your experience, can you sometimes identify a puppy (less than six months of age) that is likely to develop separation anxiety? yes, no, unsure, not applicable
